# Determinants of maternal, infant, and young child nutrition during the 1,000-day window of opportunity in Solomon Islands: A focused ethnographic study

**DOI:** 10.3389/fnut.2022.1082161

**Published:** 2023-01-19

**Authors:** Kelsey Grey, Stephen R. Kodish, Salome Aroma Namohunu, Jill Losi, Maryam Matean, Uma Palaniappan, Martina Northrup-Lyons, Arlin Cherian, Stanley Gwavuya, Judy McLean, Wendy Erasmus

**Affiliations:** ^1^Nourish Global Nutrition, Vancouver, BC, Canada; ^2^Chandlee Lab, Department of Nutritional Sciences, Pennsylvania State University, University Park, PA, United States; ^3^Chandlee Lab, Department of Biobehavioral Health, Pennsylvania State University, University Park, PA, United States; ^4^Ministry of Health and Medical Services, Honiara, Solomon Islands; ^5^United Nations Children’s Fund (UNICEF) Pacific, Suva, Fiji

**Keywords:** infant and young child feeding, Solomon Islands, Pacific Island Countries, qualitative research, MIYCN (maternal, infant, and young child nutrition)

## Abstract

**Introduction:**

This focused ethnographic study used qualitative, ethnographic, and participatory methods to explore determinants of maternal, infant, and young child nutrition (MIYCN) during the first 1,000 days of life as part of efforts to address the double burden of malnutrition in Solomon Islands.

**Methods:**

An iterative study design was used to first explore and then confirm findings related to food and nutrition security and social and behavioral determinants of MIYCN in urban and rural settings. The first phase included in-depth interviews, household observations, free lists, and seasonal food availability calendar workshops while the second phase included focus group discussions, pile sorts, participatory community workshops, and repeated household observations.

**Results and discussion:**

We found that MIYCN is shaped by a complex interaction of factors at the macro- and micro-levels. At the macro-level, globalization of the food system, a shifting economy, and climate change are driving a shift toward a delocalized food system based on imported processed foods. This shift has contributed to a food environment that leaves Solomon Islanders vulnerable to food and nutrition insecurity, which we found to be the primary determinant of MIYCN in this context. At the micro-level, this food environment leads to household- and individual-level food decisions that often do not support adequate MIYCN. Multi-sectoral interventions that address the macro- and micro-level factors shaping this nutrition situation may help to improve MIYCN in Solomon Islands.

## 1. Introduction

Malnutrition in all its forms, including undernutrition, overweight, and obesity, is the leading cause of ill health and one of the greatest challenges to sustainable development globally (1). Maternal, infant, and young child malnutrition can have intergenerational consequences for health. In the short-term, child undernutrition (i.e., stunting, wasting, micronutrient deficiencies) increases risk of morbidity and mortality, contributing to nearly half of all child deaths worldwide (2). In the long-term, maternal and child undernutrition, particularly in the first 1,000 days of life between conception and 2 years of age, can impair physical growth and cognitive development, thus reducing economic productivity and contributing to intergenerational poverty (3). Maternal malnutrition, including undernutrition and overweight and obesity during pregnancy, also increases long-term risk of non-communicable diseases (NCDs) for the child (4).

Today, the global nutrition situation is characterized by the double burden of malnutrition wherein undernutrition and overweight and obesity coexist within the same individuals, communities, and societies (5). There is a substantial burden of undernutrition among children under 5 years of age (U5), with over 149 million stunted and more than 45 million wasted. Meanwhile, nearly 39 million children U5 are obese and 2.2 billion adults are overweight or obese globally (6). Nearly half of the world’s population experiencing the double burden of malnutrition reside in Southeast Asia and the Pacific region, where the prevalence of overweight and obesity has grown faster than anywhere else in the world (7). This health crisis is driven by nutrition and food systems transitions that have increased access to processed foods high in refined carbohydrates, sugar, fat, and salt combined with reduced levels of physical activity in modern life (5).

One country experiencing the double burden of malnutrition is Solomon Islands, an archipelago of nearly 1,000 islands in the western South Pacific Ocean with an estimated population of 721,000 people in 2019 (8). Solomon Islands ranked 151 out of 189 countries in the 2020 Human Development Index, making it one of the world’s least developed countries (9). Three-quarters of Solomon Islanders live in coastal rural areas and rely on subsistence gardening and fishing for much of their food supply (8). However, the widespread availability of imported processed foods that are shelf-stable, relatively affordable, and convenient to procure and prepare is shifting traditional whole foods-based diets toward less nutritious dietary patterns (10). Maternal and child malnutrition are prevalent in Solomon Islands: 32% of children U5 are stunted, 8% are wasted, and 39% are anemic; among women of reproductive age, 48% are overweight or obese and 54% of pregnant women are anemic (11).

Population-level nutritional status is determined by a combination of immediate, underlying, and enabling factors (12). An important underlying factor is infant and young child feeding (IYCF) practices, which include breastfeeding and complementary feeding. Although approximately 75% of infants are exclusively breastfed until 6 months in Solomon Islands, sub-optimal complementary feeding practices persist. Only 22% of children aged 6–23 months are fed according to IYCF recommendations for meal frequency and diversity and less than half receive iron-rich foods (11). Optimal IYCF practices rely on household food and nutrition security. However, in Solomon Islands, food and nutrition security challenges are widespread. In both rural and urban areas, approximately half of total household consumption expenditure goes toward food, indicating at least a moderate level of food and nutrition insecurity among most households (13, 14).

While previous nutrition surveys have revealed sub-optimal nutrition indicators in Solomon Islands, a deeper understanding of the behavioral factors influencing diets has been much less reported (15, 16). Therefore, this study was designed to: (1) understand food availability and accessibility across seasons, (2) describe the underlying social and behavioral determinants of maternal, infant, and young child nutrition (MIYCN) in the first 1,000 days of life in urban and rural settings, and (3) generate context-specific recommendations to inform appropriate social and behavior change communication strategies for improved MIYCN, health, and survival in Solomon Islands. These objectives were aligned with MIYCN strategies outlined in the Solomon Islands National Food Security, Food Safety, and Nutrition Policy for 2010–2015, the available policy at the time of this study (17).

## 2. Materials and methods

### 2.1. Study setting

**FIGURE 1:**
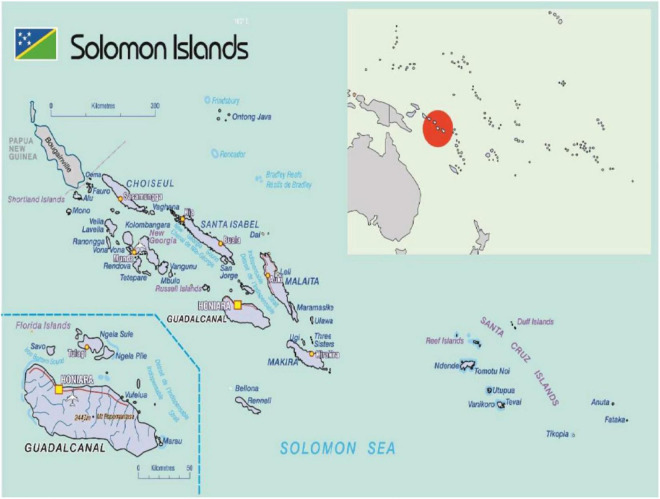
Map of Solomon Islands within the Pacific region, highlighting Guadalcanal Province in blue and Honiara in yellow (bottom left) (11).

Data collection took place in both urban and rural communities of Solomon Islands between May and June 2018. The data collection sites were selected with the Solomon Islands Ministry of Health and Medical Services (MHMS) and UNICEF based on criteria to allow for adequate sample sizes across methods and participant types (Figure 1).

#### 2.1.1. Urban data collection: Kola’a (Honiara)

Urban data collection occurred in Kola’a, one of 12 wards in central Honiara, the capital city of Solomon Islands located on Guadalcanal, the country’s largest island by geographic area (11). Honiara is densely populated with approximately 5,950 people per square kilometer (8). Nine communities from Kola’a ward were selected for this study. Due to increasing urbanization, many households in Honiara are shifting from agriculture to more service-oriented livelihoods (18). While some households make efforts to grow vegetables in their backyard gardens or raise livestock or fish in small scale, most people in Honiara buy produce and fish at Honiara Central Market (10). People living in Kola’a ward have easy access to imported foods high in carbohydrates and fat given its proximity to city markets and the presence of roadside shops in sub-urban neighborhoods. The consumption of imported foods such as rice and flour-based foods is higher in Honiara compared to more rural parts of Solomon Islands (10, 11).

#### 2.1.2. Rural data collection: Malango (Guadalcanal Province)

Rural data collection took place in Malango ward in central Guadalcanal Province, located approximately 40 km southeast of Honiara. Guadalcanal Province is the largest province of the Solomon Islands by geographic area with an estimated population of 154,150 in 2019. Population density is low at 29 people per square kilometer (8). Thirteen communities from Malango were selected for this study (19). This area lies in the northern coastal plains of the island where optimal rainfall patterns and fertile soil create strong growing conditions. Subsistence gardening provides for most household food intake in this area, and households earn income by selling surplus produce in Honiara’s markets. The consumption of imported foods like rice and flour-based products is high compared to other rural areas of Solomon Islands given its proximity to Honiara (10).

### 2.2. Study design

This study used an iterative, mixed methods design drawn from Focused Ethnographic Study procedures (20). Data were collected over two phases: Phase 1 was exploratory in nature to identify emergent themes and Phase 2 was confirmatory to corroborate, clarify, and build on findings from Phase 1. Phase 1 data was analyzed before commencing Phase 2 and the findings informed Phase 2 data collection instruments (Table 1).

**TABLE 1:** Study design with data collection methods by study phase.

**Phase 1: Exploratory**	Phase 1 data analysis to inform Phase 2 instruments	**Phase 2: Confirmatory**
● In-depth interviews		● Focus group discussions
● Free lists		● Pile sorts
● Seasonal food availability calendar workshops		● Participatory community workshops
● Household observations		● Household observations

### 2.3. Data collection methods and sampling procedures

Ten locally hired data collectors were selected based on previous experience conducting qualitative nutrition or health-related fieldwork, education level, computer literacy, proficiency in English and Pidgin (the local lingua franca), and previous experience with translation or transcription.

#### 2.3.1. Phase 1: Exploring determinants of MIYCN

In-depth interviews (*n* = 51) were conducted in Phase 1 among caregivers of children aged 6–23 months (e.g., mothers, fathers, grandparents), community leaders (e.g., elected officials, religious, and traditional leaders), community-level health workers (e.g., nurses, community health volunteers, traditional healers), and senior-level health staff (e.g., district-level or national-level health staff from the MHMS). Participants were asked a series of semi-structured questions covering MIYCN, child health, food security, water, sanitation, and hygiene practices, gender and family roles, and preferred communication channels. Semi-structured interview guides were tailored to each participant type to ensure that questions were appropriate and relevant. Interviews lasted 45–60 min and were conducted in Pidgin, except for those among senior health staff which were conducted in English.

Free lists (*n* = 89) were conducted with caregivers to elucidate salient young child foods and illnesses specific to the cultural context of Solomon Islands. Free listing is a cognitive anthropology method used to elicit salient items of a cultural domain (i.e., a local body of knowledge pertaining to a specific topic) (20).

Seasonal food availability calendar workshops (*n* = 2) were conducted to understand the seasonal nature of food availability. Participants included farmers, food vendors, and consumers who were tasked with creating a calendar outlining foods available by season in their community. For each food, participants indicated (1) no availability, (2) low availability, (3) medium availability, or (4) high availability. A final discussion was held to reach consensus over accuracy of the calendar.

Household observations (*n* = 18) were conducted to gain an understanding of intra-household factors that influence IYCF. Full-day observations (i.e., for 10–12 h from the child’s first meal until last meal) focused on breastfeeding and complementary feeding practices as well as hygiene behaviors. A semi-structured form was used to document behaviors and events at least every 10 min. These observations were repeated among the same households and children in Phase 2 to reduce reactivity to the presence of the data collectors (21).

#### 2.3.2. Phase 2: Confirming and consolidating factors influencing MIYCN

Focus group discussions (*n* = 8) were conducted among caregivers of children aged 6–23 months to identify social norms around MIYCN and to triangulate interview findings. Focus groups were conducted separately for male and female caregivers.

Participatory community workshops (*n* = 4) were held with diverse community members who were selected to represent the community. During workshops, participants brainstormed, and voted upon top-ranked barriers to optimal MIYCN in each setting as well as suggested intervention strategies to overcome those barriers. Community workshops have been used successfully to engage communities in identifying priority intervention areas and to develop culturally relevant messaging as part of participatory research (22, 23).

Pile sorts (*n* = 81) were conducted among caregivers to assess how and to what degree salient young child foods and illnesses identified during free listing were perceived to cluster together, thus revealing local food and illness classification systems (24). Salient free list items (i.e., the top free listed terms scoring ≥ 0.30 (*S*), which is calculated by both frequency and order of items mentioned) were written on cards for sorting into piles based on question prompts.

### 2.4. Sampling

The Social Ecological Model (SEM), which acknowledges multi-level factors of behavior, served as a guiding theoretical framework for this study and framed our purposive sampling approach (25) (Table 2).

**TABLE 2:** Participant types by level of the Social Ecological Model (SEM).

Level of influence	Participant types
Policy	Senior-level health staff (e.g., MHMS nutrition staff, agricultural extension officers)
Organizational	Professional health workers (e.g., nurses, midwives)
Community	Community leaders (e.g., religious leaders, village leaders, youth leaders)
Interpersonal	Fathers, grandparents
Individual	Primary caregivers (typically mothers)

Next, a criterion-based sampling approach was used to identify specific types of participants within each behavioral level. Local health workers with knowledge of the community then assisted with recruitment of eligible participants. Sample sizes for interviews, focus groups, and direct observations were based on the estimated amount of textual data needed to reach “data saturation” in key areas of inquiry (26). Samples sizes for free lists and pile sorts were selected to ensure validity of cultural domain analysis, as recommended by Weller and Romney (27). Community workshop sizes were chosen to allow for comfortable facilitation of participatory methods and based on previous research using this method (22, 28) (Table 3).

**TABLE 3:** Sample sizes by data collection method, participant type, and study site.

Data collection method	Sample size (*n*)		
	**Urban**	**Rural**	**Total**
In-depth interviews	26	25	51
Female caregivers	10	10	20
Male caregivers	5	5	10
Community leader	5	5	10
Health worker	6	5	11
Senior health staff*^a^*	–	–	4
Pile sorts	40	41	81
Free lists	45	44	89
Focus group discussions	4	4	8
Female caregivers	2	2	4
Male caregivers	2	2	4
Community workshops	2	2	4
Female community members	1	1	2
Male community members	1	1	2
Household observations	10	8	18
Children aged 6–11 months	5	3	8
Children aged 12–23 months	5	5	10
Seasonal food availability calendar workshop	1	1	2

^a^Sampling for senior health staff interviews was not designated urban or rural as the staff worked in both sites.

### 2.5. Data analysis

#### 2.5.1. Textual data analysis: Interviews, focus groups, direct observations

Interviews and focus group discussions were conducted in Pidgin and recorded using digital audio recorders. Locally hired team members then simultaneously transcribed and translated the audio files into English. Themes pertinent to the study objectives were identified across transcripts in line with Grounded Theory (29, 30). These themes were then synthesized in an analytic codebook that reflected both the interview guide content and any newly identified themes. The codebook was used to guide systematic identification and labeling of relevant themes across transcripts using NVivo 12 software (31). The coded text was stratified by participant type and method and extracted in relation to each research question. The results were confirmed through triangulation with findings from other study methods.

#### 2.5.2. Cultural domain analysis: Free lists and pile sorts

Free list items for each participant were analyzed using Anthropac 4.98 software (32). A salience statistic was calculated for each item based on its rank order (24, 27). Young child foods and illnesses identified as salient were interpreted in relation to interview findings for a more complete ethnographic perspective. Free list items were chosen for inclusion in the pile sort activity if they had a salience greater than 0.30 or were deemed relevant to addressing the study aims; for example, breastmilk was not identified as a salient “young child food” in free lists but was included in pile sorts due to its nutritional importance. Pile sort data were then entered into Anthropac 4.98, which calculated aggregate proximities and generated item-by-item matrices for items with cells indicating the proportion of times two items appeared in the same pile across participants. Multi-dimensional scaling was used to analyze those aggregate proximity matrices (27). The goodness-of-fit, or stress, was also calculated for each matrix and is indicative of the strain remaining when the items in a cultural domain have been fitted into two dimensions. Stress values range from 0 (worst fit) to 1 (best fit). Two-dimensional visual maps were generated, and classifications were labeled based on field notes.

#### 2.5.3. Workshop analysis: Seasonal food availability and community workshops

Seasonal food availability workshop data were entered into a customized template. Symbols used to represent the relative availability of foods across seasons were translated into numerical values following standardized analytic procedures used elsewhere (33, 34). The food items listed by workshop participants were grouped according to local food classification systems derived from pile sort results (e.g., “body-building,” “protective,” and “energy” foods) to create a color-coded calendar depicting seasonal food availability across the year. The numerical data from other participatory community workshops were analyzed using simple arithmetic to tally and rank the top-voted barriers and intervention strategies.

### 2.6. Ethical considerations

Ethical approval was obtained from the ethics committee of the Solomon Islands MHMS. The procedures used in this study adhere to the tenets of the Declaration of Helsinki. Participants’ oral informed consent was obtained by the data collectors prior to any data collection.

## 3. Results

### 3.1. Determinants of food availability and food accessibility

Household food insecurity challenges underlie the nutrition situation in Solomon Islands. Downstream, individual-level dietary decisions in Solomon Islands are determined by upstream factors related to food availability and accessibility throughout the year.

#### 3.1.1. Food availability

In Kola’a (urban site), a variety of food groups are available across seasons (Table 4). However, urban households produce only a small proportion of their own food for consumption and rely on cash income to purchase foods available in markets. Locally produced fresh foods are primarily sold in a limited number of markets along the main road in Honiara. Fresh food items can also be found in small roadside shops outside of the main road but are sold at a substantial mark-up and can be difficult to access.

**TABLE 4:** Seasonal food availability calendar for Kola’a (urban site).

	Jan	Feb	Mar	Apr	May	Jun	Jul	Aug	Sept	Oct	Nov	Dec
**Seasons**	**Heavy rain**				**Partly dry season**							
**Food**												
**Energy Foods**												
Cassava*												
Potato*												
Yam*												
Lesser yam (*pana*)*												
Rice												
Noodles												
Bread products												
Taro*												
Coconut (green and mature)*												
Butter												
**Body-building Foods**												
Fish (fresh)*^a^												
Fish (salted)*^b^												
Tuna (tinned)*												
Milk												
Peanut*												
Chicken												
Sausage												
Minced meat (beef)												
Shellfish*												
Mud crab*												
Crab*												
**Protective Foods**												
Banana (sweet and green varieties)*												
											
Slippery cabbage*^c^												
Melon*												
Cucumber*												
Pineapple*												
Malay apple*												
Tomato*												
Eggplant*												
Onion												
Pumpkin*												
White bean*^d^												
Watercress*												
Fern*												
Pumpkin shoots/leaves*												
Taro leaf*												
Chinese cabbage*												
Mandarin orange*												
Mangrove fruit*												
**Other**												
Sugar												
Tea												
Juice												
Betel nut*												

High availability 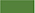; Medium availability 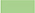; Low availability 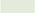; No availability 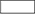.

* Denotes locally produced foods.

^a^Weather (rough seas) affects the availability of fresh fish.

^b^Availability of salt fish drops off as fishing boats go for on-shore maintenance during fish spawning season from August to December.

^c^Slippery cabbage, scientifically known as Abelmoschus manihot (L.) Medic., is a dark green vegetable.

^d^White bean, locally known as “bean,” has the appearance of a melon. Young fruit of the white bean is cooked before consumption. Blue colored indicate the “Rainy season”. Orange colored indicate the “Dry season”.

*“Most of the time we eat processed food rather than local food*… *because we don’t have a garden here and most of the time we don’t go to the market because it’s far from us.”*

– Female caregiver interview, Kola’a (urban)

A wide variety of foods was also found to be available across seasons in Malango (rural site) (Table 5).

**TABLE 5:** Seasonal food availability calendar for Malango (rural site).

	Jan	Feb	Mar	Apr	May	Jun	Jul	Aug	Sept	Oct	Nov	Dec
**Seasons**	**Rainy**	**Sun**			**Rain and sun**	**Dry season**						
**Food**												
**Energy Foods**												
Cassava*												
Potato*												
Lesser yam (*pana*)*^a^												
Yam*												
Taro*												
Rice												
Noodle												
Coconut (dry, mature)*												
Bread products (cake)^b^												
Biscuit												
Breadfruit*												
Coconut (green)*												
Sugar cane*												
Popcorn												
**Body-building Foods**												
Tuna (tinned)*												
Chicken*^c^												
Fish (salted)*												
Ngali nut*												
Cut nut*^d^												
Shell fish (from the river)*												
Peanut*												
Pork*												
Fish (fresh, from the river)*												
**Protective Foods**												
Banana (sweet and green)*												
Jack fruit*												
Fern*												
Eggplant*												
Tomato*												
White bean*												
Guava*												
Watercress*												
Taro leaf*												
Pineapple*												
Cucumber*												
Papaya*												
Pumpkin*												
Chinese cabbage*^e^												
Choy sum (dark leafy green)*^f^												
Soursop*												
Lemon*												
Sweet leaf (*bonio*)*												
Inkori*^g^												
Avocado*												
Malay-apple*												
Apple												
Mango*												
**Other**												
Sugar												
Salt												
Coffee mix												
Betel nut*												
Candy (lollipops)												

High availability 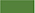; Medium availability 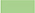; Low availability 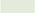; No availability 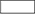.

* Denotes locally produced foods.

^a^A variety of root crop, locally known as pana.

^b^The availability of bread products in the rural area depends on whether they are locally baked, and access to grocery stores or bakeries in the urban area.

^c^The availability of chicken is low throughout the year in the rural area as chicken is primarily raised for sale in Honiara markets.

^d^Cut nut, scientifically known as Barringtonia novae-hibernae Laut., is eaten fresh or roasted, are milky in taste and have a hard texture.

^e^Chinese cabbage is also referred to as bok-choy; it has white stems and dark green leaves.

^f^A type of Chinese cabbage with long green stem and green leaves.

^g^A local fruit from the Santa Isabel province of the Solomon Islands. It is similar in appearance to a pear, has the crunchiness of an apple, and tart flavor. Blue colored indicate the “Rainy season”. Orange colored indicate the “Dry season”. Yellow colored indicate the “Sun”. Green colored indicate the “Mix of rain and sun”.

Compared to urban households, more rural households engage in home gardening, conserving root crops, and cabbage for consumption while selling fruits and vegetables in urban markets. Participants explained that income made from selling produce is typically used to purchase processed foods such as rice and canned tuna.

*“For those of us working in the garden, we plant slippery cabbage and then we harvest it to sell at the market*… *For me, when I sell food at the market, I usually get $100 or even $200 [Solomon] dollars in a day. We do not eat the food that we plant; we sell it at the market and when we return home we buy rice, bread, sugar*,…*and the money goes again.”*

– Seasonal food availability workshop, Malango (rural)

Factors that influence this decision include: (1) the greater value for money provided by processed foods, (2) the relative convenience of procuring, storing, and preparing processed foods, (3) a taste preference for processed foods, and (4) the safety net that shelf stable foods provide if garden crops fail.

Observations found that fresh fish and meat are not sold in the small dry goods shops that are found throughout rural communities. Although some rural households raise chickens or pigs, these animals are usually sold for income and only eaten during special occasions such as weddings.

In both study sites, participants explained that increasing homestead food production has been hampered by agricultural challenges including climatic events (e.g., drought, flooding, cyclones), land shortages, especially in urban areas, and crop damage caused by Giant African Snails, a recently introduced invasive species.

#### 3.1.2. Food accessibility

Although nutritious local foods are available throughout the year in Kola’a (urban) markets, high food costs and large family sizes relative to household incomes limit local food access, contributing to a reliance on processed food imports.

*“For those living in the urban area, local foods are very expensive at the market and then we have the problem of the middle-men or middle sellers. The re-sellers buy [food] at cheap prices and re-sell it at a higher price so that makes it more difficult for urban people to get those healthy foods, like local foods, for their family.”*

– Senior national health staff, Ministry of Health and Medical Services

Fruits and vegetables are comparatively more accessible in Malango (rural) where home gardening is practiced. However, limited access to animal-source foods remains a challenge for most households. Opportunities to access fresh fish are limited in the inland rural area due to its distance from the coast and access to canned tuna in local shops is limited by low household incomes.

*“There are so many things we want to eat, especially for those of us living in the bush far from the sea*…*fish is one of the foods that we really love to eat but we can’t afford it because it’s too expensive – not canned tuna, but the kind that they sell in Eskys [large coolers] that you can buy by the pound. We go to town [Honiara] to buy fish but most of the time it is too expensive.”*

– Male caregiver, Malango (rural)

**FIGURE 2:**
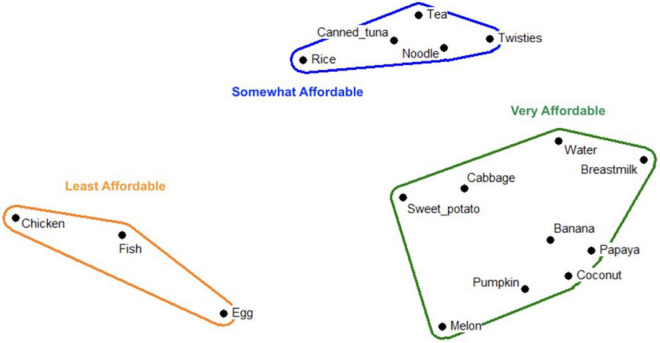
Multi-dimensional scaling map depicting affordability of child foods in Kola’a (urban site); Stress: 0.093; Eigen value: 8.427; Eigen ratio: 3.243.

**FIGURE 3:**
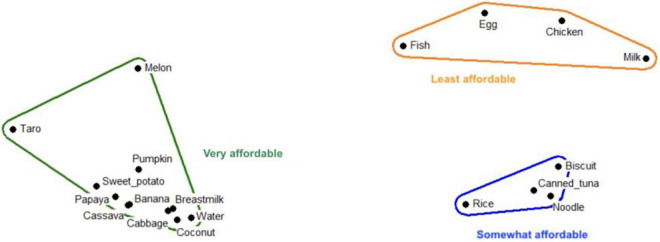
Multi-dimensional scaling map depicting affordability of child foods in Malango (rural site); Stress: 0.042; Eigen value: 13.264; Eigen ratio: 3.025.

The unaffordability of animal-source foods emerged as a key finding stemming from multiple methods including pile sorting (Figures 2, 3).

#### 3.1.3. Typical family meals

In Kola’a (urban), observations revealed typical meals consisting of rice, canned tuna, and noodles for all household members older than 12 months of age. Common breakfast foods include bread or biscuits (i.e., cookies) eaten with *tea* (refers to different combinations of hot water with lemon leaf, black tea, instant coffee, malt drink mix, sugar, and milk). In Malango (rural), typical meals consist of rice, potato, or cassava eaten with a soup of dark leafy greens (e.g., taro leaf, ferns) boiled in coconut milk. Rice may be eaten alone or with soup for breakfast along with *tea*. During mealtimes, it is a social norm for male heads of household and visitors (e.g., extended family) to be served foods before mothers and children in both Kola’a and Malango.

### 3.2. Factors influencing MIYCN in the first 1,000 days of life

#### 3.2.1. Maternal diets during pregnancy and lactation

In both Kola’a and Malango, pregnant women eat typical family foods despite awareness that local foods (e.g., cabbage, fruits, potato, fish) may provide a more nutritious diet than processed foods. Health workers explained that although they encourage pregnant and lactating women to consume more local foods and avoid salty, oily, and sugary foods, maternal diets are primarily governed by the household’s level of food access. Women also described receiving dietary advice from husbands and elders who encourage consumption of local foods, “*to increase or strengthen the blood*” (i.e., prevent anemia) in preparation for childbirth.

*“The very important thing about eating fruits and vegetables is that when they [mothers] reach the time for delivery, bleeding will be high during that time, and it will be a risk for them. So, fruits and vegetables will prepare them so that their blood becomes strong, so that they can produce more blood*…*so when they lose blood there’s enough to survive during delivery.”*

– Male caregiver focus group, Kola’a (urban)

Interviews revealed food rules specific to these life stages that influence dietary choices among mothers. During pregnancy and lactation, large deep-sea fish (e.g., tuna, barracuda) are proscribed (taboos) as they are said to cause “*fish sick”* in the unborn or breastfeeding infant due to their high sodium content. “*Fish sick”* was described as a mouth rash resembling oral thrush and most often ascribed to “*bonito”* consumption (tuna caught by offshore trawlers and stored in sea water).

*“We have heard that breastfeeding women should not eat too much fish from the deep sea like bonito [tuna] because it can cause sores in the baby’s mouth.”*

– Female caregiver focus group, Malango (rural)

Food prescriptions (remedies) during lactation were also identified. Mothers are encouraged to increase fluid intake and to consume coconut milk soups made with cabbage, fish, and potato to increase breastmilk production. *Gheke*, a locally available cabbage with yellow leaves, is said to increase breastmilk production, while “dry foods,” such as rice and roasted potatoes, are said to limit production.

#### 3.2.2. Breastfeeding

Overall, participants described positive breastfeeding practices in line with global recommendations. The initiation of breastfeeding within an hour of birth is common in urban and rural areas as reported by both mothers and health workers. Exclusive breastfeeding until 6 months was reported to be more widely practiced than in the past when foods and liquids were more commonly introduced early due to traditional perceptions that doing so accelerated infant growth.

*“The belief for those in the past is that they do not wait for the baby to reach 6 months of age to feed a baby with liquids or solid foods. They fed their baby usually at 2 or 3 months*…*or even 2 weeks, they gave water or juice.but nowadays, the nurses or doctors advise us to feed our child at 6 months.”*

– Female caregiver focus group, Malango (rural)

However, two important barriers to exclusive breastfeeding remain. First, working mothers, primarily in Kola’a, explained that exclusive breastfeeding is disrupted during work hours when their infants are under the care of alternative caregivers. Second, perceptions of inadequate breastmilk supply were reported in both Kola’a and Malango.

While most mothers reported continuing breastfeeding beyond 6 months of age, they described discontinuing the practice upon becoming pregnant due to the perception that breastfeeding during pregnancy causes diarrhea in the breastfeeding child and that breastmilk quality is reduced.

#### 3.2.3. Complementary feeding

Caregivers reported introducing some fruit juices and watery foods (e.g., pawpaw juice, pumpkin softened with water) when infants reach 6 months of age. Typically, caregivers delay the introduction of semi-solid foods until 8–10 months of age due to the perception that younger infants are not developmentally ready to digest these foods.

*“Still, the mothers, even though they say they start complementary feeding at 6 months, they still start using the pawpaw juices because they say that the child’s stomach or the digestive system is not ready to accept the food yet*…”

– National health staff, Ministry of Health and Medical Services

Infants aged 6–11 months are typically fed specially prepared meals of watery, mashed foods (e.g., pawpaw, potatoes, pumpkin) or juices (e.g., pawpaw juice) three times daily, with snacks between meals (e.g., bananas, biscuits softened with *tea*) in addition to continued breastfeeding.

*“For me, in the morning I boil a pawpaw fruit. After it is boiled or cooked, I mash it and give the pawpaw juice to my child. In the afternoons, I do not boil the pawpaw but just mash it and give its juice to the baby. In the evening, I also cook or boil pawpaw and then mash it and give the juice to the baby.”*

– Female caregiver interview, Kola’a (urban)

Dietary diversity remains a relatively greater challenge for infants aged 6–11 months than for children 12–23 months, given the normative practice of introducing foods at 8–10 months. Consumption of “deep-sea fish” is culturally proscribed for infants, just as it is for pregnant and lactating mothers.

At 12–23 months, young children begin sharing family foods and breastfeed less frequently. Meal observations found young children to frequently consume sugar-sweetened beverages (e.g., *tea*) and imported processed food (e.g., candy, biscuits) in both study sites. Caregivers generally reported adequate knowledge of nutritious versus non-nutritious foods, but low household income relative to food costs and greater availability of processed foods underlie feeding decisions.

*“In Solomon Islands, children eat carbohydrates as their normal diet. Some children have access to fruits while some do not at all because the cost of living is too expensive. Only rice is cheap ($10.00 per kg) and can feed the whole family, while buying a $10.00 heap [fruits and vegetables are sold in small heaps] at the market can only fed two or three family members*…*most children don’t have access to a balanced diet.”*

– Nurse interview, Kola’a (urban)

During participatory workshops, community members brainstormed and voted upon the top challenges to and solutions for improving infant and young child nutrition (Tables 6, 7). Limited community awareness of the importance of optimal child nutrition and a preference for processed foods emerged as the top challenges in Kola’a and Malango, respectively.

**TABLE 6:** Challenges and solutions for improving infant and child nutrition in Kola’a (urban).

# votes	Top-voted challenges	Top-voted solutions
27	Little awareness in the community and clinics about nutrition, balanced diet, and health information	Education around balance diets will help families to eat a proper diet
25	Lack of improved sanitation facilities (water systems, toilets), clean and safe drainage systems, and hygiene practices	Need financial support from potential donors to build proper water systems and sanitation facilities
11	Too many children in households	Need better family planning to have more money available to support the needs of a household

**TABLE 7:** Challenges and solutions for improving infant and child nutrition in Malango (rural).

# votes	Top-voted challenges	Top-voted solutions
19	People prefer processed foods over local foods	Self-discipline to not buy processed foods
14	Lack of knowledge among the community on balanced diet	Educate our children on the importance of a balanced diet
8	Poor hygiene practices such as no handwashing after toilet use, no breast washing after gardening, no handwashing after cleaning the household or the environment, not cleaning child’s dirty clothes, no proper covering of foods from flies.	Train mothers on proper hygiene

## 4. Discussion

Like many Pacific Island Countries, Solomon Islands is facing multiple threats to food and nutrition security that are contributing to a growing burden of multiple forms of malnutrition (7, 35). Our results provide insight into the nutrition situation of urban and rural island settings where local diets are shaped by both micro- and macro-processes.

Although Solomon Islands has diverse foods available across seasons, the modern-day food environment is characterized by locally produced fresh foods that are more difficult to access than most imported processed foods. At a macro-level, globalization and related economic policies have helped to shape this delocalized food system where energy-dense, non-nutritious dietary patterns are now prominent (15, 16). We found that most families prefer imported foods because they are more affordable, convenient, and palatable despite awareness that they are less nutritious. In rural Malaita and Western provinces of the country, where Albert et al. found that just 6% of women consumed a minimum number of food groups each day, study participants acknowledged that typical diets reliant on imports such as rice, noodles, and processed meats were the main community health problem (15). Similarly, a mixed methods study conducted by Vogliano et al. in a remote community in Western province found that over two-thirds (73%) of participants did not meet the requirements of the Minimum Dietary Diversity Score for Women, with ease of access and convenience of imported foods described as drivers of historical food system transitions away from traditional diets (16). Given the critical role that locally produced fresh foods play in supporting maternal health and child development, promoting local agricultural production and traditional food production practices, strengthening social marketing of local foods, and discouraging the consumption of non-nutritious processed foods are key actions to improve nutrition outcomes in Solomon Islands. These actions are included as key policy areas for action in the Solomon Islands’ updated National Food Security, Food Safety, and Nutrition Policy for 2019–2023 released since the completion of this study (36).

Knowledge is necessary but not sufficient for nutrition-related behavior change, especially in Solomon Islands where a shift to a market-based economy has contributed to new food and nutrition security challenges (15, 37). In the past, Solomon Islanders primarily accessed food through fishing, subsistence gardening, hunting, and trading; however, the socio-economic shift toward service-based industries and exports has altered food access patterns, and consequently, consumption (15, 38). For example, we found rural households engaging in homestead food production not for their own consumption, but to generate sales whose profits can be used to buy imported foods that more easily meet the caloric needs of a large family. As incomes rise, rural households tend to purchase more imported foods in this setting (16). In urban Kola’a, caregivers explained that the opportunity cost of being employed outside of the home, which is now more commonplace, means inadequate time available for producing foods at home.

While nutrition-sensitive interventions, such as those promoting home gardens to increase household vegetable consumption, have positively improved local diets in other settings, Solomon Islands offers a challenging context for local food production given its vulnerability to climate-related threats (39). Rising ocean levels and temperatures, more frequent droughts and storms, greater flooding due to heavier rains, and increased pests have made local food production through gardening and fishing increasingly difficult in this setting (16, 40). In fact, participants in our study specifically ascribed recent crop failures to drought and flooding as well as damage caused by Giant African Snails. Research in coastal areas of the country has attributed changing oceanic conditions to falling fisheries production (38). These vulnerabilities may help to explain the reliance on imported foods in Solomon Islands.

While investigating maternal diets, we found that the diets of pregnant and lactating women are similar to those consumed by other adults given the general challenges to accessing more nutritious foods. In some cases, however, data suggest that cultural food rules (e.g., avoidance of certain fish during pregnancy; increased consumption of fluids during lactation) may influence maternal diets in this setting. In Solomon Islands, where consumption of processed foods high in sodium is commonplace, avoiding proscribed foods such as “deep-sea fish” may offer some protection against excess sodium intake and risk of hypertension, for instance (41). Similarly, increased fluid intake during lactation may have some beneficial effects given the role of adequate hydration in milk production (42, 43).

National survey data corroborate our findings that breastfeeding practices are strong among Solomon Islanders. Most mothers initiate breastfeeding within 1 h of birth (79%) and exclusively breastfeeding for 6 months (75%) in this setting where past initiatives have shaped an enabling environment for breastfeeding (11). For instance, the national referral hospital in Honiara has joined the Baby Friendly Hospital Initiative, which promotes optimal care for new mothers (44). At the community level, professional health workers promote breastfeeding, an approach that has been effective in other settings where care groups and similar interpersonal support are built into the health system. Strengthening maternity protection legislation by enacting gender-sensitive workplace policies (e.g., compulsory lactation rooms in work places, paid breastfeeding breaks) may specifically help the mothers in our study who reported breastfeeding challenges while working (45). Given the island-wide burden of NCDs, continued investment in breastfeeding initiatives may be a cost-effective approach to help mitigate disease risk even into adulthood (46).

Finally, we observed infant and young child diets characterized by frequent consumption of both sugar sweetened beverages and snacks (e.g., biscuits) and low dietary diversity. While more than one third (37%) of Solomon Islands children under 2 years consumed adequately diverse diets in 2015, just 14% of those aged 6–8 months did so (11). Community members suggested education-based interventions for caregivers, as well as financial support and family planning approaches, to help families improve child nutrition. Given the complex upstream factors that shape this food environment, interventions to improve child nutrition in Solomon Islands may benefit from multi-level prevention strategies targeting modifiable aspects of the food environment above and beyond the individual level. For instance, intervening through small food stores where unhealthy snack foods are often purchased has shown promise in other Pacific Island Countries and beyond (47, 48).

We designed this study to be iterative, with multiple phases of data collection that allowed for findings from one phase to inform the next. Such a design is an important strength of this type of exploratory qualitative research using mixed ethnographic methods. Another aspect of our study that gives us confidence in our findings is the methodological triangulation used to answer research questions from different perspectives. Triangulation is an established strategy for helping to ensure data credibility in this type of research (49). Our study also has several limitations that should inform interpretation of our findings. Firstly, rural data collection occurred in Malango, a site in the fertile northern plains of Guadalcanal province with ideal growing conditions and good market access, thus limiting our ability to extrapolate findings to other rural islands (10). Secondly, data collection occurred during one season of the year when findings may be season specific. However, we tried to account for seasonality by using specific methods like the seasonal food availability workshops for data generation across the calendar year. Finally, while we conducted repeated meal observations among the same households to reduce reactivity it is likely that the presence of observers may have altered some feeding behaviors of interest (21).

## 5. Conclusion

Our study revealed that MIYCN in the first 1,000 days of life in Solomon Islands is shaped by a complex interplay of micro- and macro-level factors that result in food and nutrition insecurity at the household level, thus negatively impacting maternal diets and complementary feeding practices. Given the complexity of these interacting factors, a multi-sectoral approach addressing all behavioral levels of influence may help to improve the nutrition situation of women and children in Solomon Islands.

## Data availability statement

The raw data supporting the conclusions of this article will be made available by the authors, without undue reservation.

## Ethics statement

The studies involving human participants were reviewed and approved by the Solomon Islands Ministry of Health and Medical Services. The ethics committee waived the requirement of written informed consent for participation.

## Author contributions

WE, UP, and SG: conceptualization. SK, MN-L, and JM: methodology. MM, KG, and MN-L: formal analysis. KG, MM, MN-L, AC, SN, and JL: investigation. KG and SK: writing—original draft preparation. All authors read and approved the final manuscript.
